# Supplementary material for: Causal Relationship between Aging and Anorexia Nervosa: A White-Matter-Microstructure-Mediated Mendelian Randomization Analysis
Source: Biomedicines. 2024 Aug 16;12(8):1874. doi: 10.3390/biomedicines12081874 (PMC11351342; doi:10.3390/biomedicines12081874)
Supplement: Supplementary file 1 [file biomedicines-12-01874-s001.zip › Supplementary Figure/Figure S8/Leaveoneout plot of MR analysis for TL to ubm-b-1547.pdf]

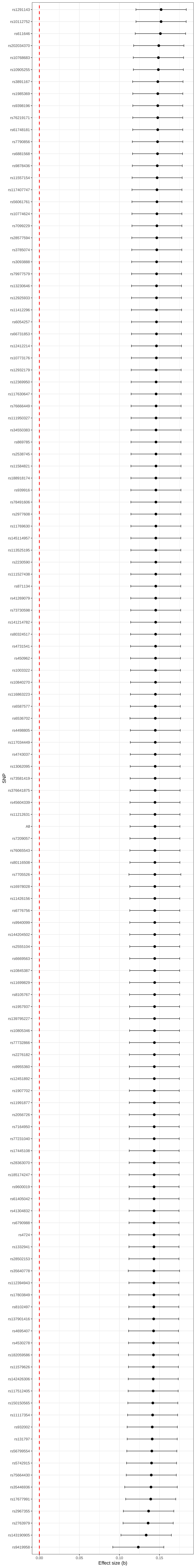

SNP
Effect size (b)
0.00
0.05
0.10
0.15
rs1291143
rs10112752
rs611646
rs202034370
rs10768683
rs10905255
rs3891167
rs1985369
rs9398196
rs76219171
rs61748181
rs7790856
rs6881568
rs9878436
rs11557154
rs117407747
rs56061761
rs10774624
rs7099229
rs28577594
rs3785074
rs3093888
rs79977579
rs13230646
rs12925933
rs11412296
rs6054257
rs66731853
rs12412214
rs10773176
rs12932179
rs12369950
rs117630647
rs76666449
rs111950327
rs34550383
rs869785
rs2538745
rs11584821
rs188918174
rs939916
rs78491606
rs2977608
rs11769630
rs145114957
rs113525195
rs2230590
rs111527438
rs871134
rs41269079
rs73730598
rs141214782
rs80324517
rs4731541
rs450962
rs1003322
rs10840270
rs116863223
rs6587577
rs6536702
rs4498805
rs117034449
rs4743037
rs13062095
rs73581419
rs376641875
rs45604339
rs11212631
A8
rs7209057
rs76065543
rs80116508
rs7705526
rs16978028
rs11426156
rs6776756
rs9940099
rs144204502
rs2555104
rs6669563
rs10845387
rs11699829
rs8105767
rs1957937
rs139795227
rs10805346
rs77732866
rs2276182
rs9955360
rs12451892
rs1907702
rs11991877
rs2056726
rs7164950
rs77231040
rs17445108
rs28363070
rs185174247
rs9600019
rs61405042
rs41304832
rs6790988
rs4724
rs1332941
rs28502153
rs35640778
rs112394943
rs17803849
rs8102497
rs137901416
rs4695407
rs4530278
rs182059586
rs11579626
rs142426306
rs117512405
rs150150565
rs11117354
rs932002
rs131797
rs56799554
rs5742915
rs75664430
rs35446936
rs17677991
rs2967355
rs2763979
rs143190905
rs9419958
